# Exploring the impact of network depth on 3D U-Net-based dose prediction for cervical cancer radiotherapy

**DOI:** 10.3389/fonc.2024.1433225

**Published:** 2024-09-16

**Authors:** Mingqing Wang, Yuxi Pan, Xile Zhang, Ruijie Yang

**Affiliations:** Department of Radiation Oncology, Cancer Center, Peking University Third Hospital, Beijing, China

**Keywords:** 3D U-Net, dose prediction, radiotherapy, network depth, cervical cancer

## Abstract

**Purpose:**

The 3D U-Net deep neural network structure is widely employed for dose prediction in radiotherapy. However, the attention to the network depth and its impact on the accuracy and robustness of dose prediction remains inadequate.

**Methods:**

92 cervical cancer patients who underwent Volumetric Modulated Arc Therapy (VMAT) are geometrically augmented to investigate the effects of network depth on dose prediction by training and testing three different 3D U-Net structures with depths of 3, 4, and 5.

**Results:**

For planning target volume (PTV), the differences between predicted and true values of D_98_, D_99_, and Homogeneity were statistically 1.00 ± 0.23, 0.32 ± 0.72, and -0.02 ± 0.02 for the model with a depth of 5, respectively. Compared to the other two models, these parameters were also better. For most of the organs at risk, the mean and maximum differences between the predicted values and the true values for the model with a depth of 5 were better than for the other two models.

**Conclusions:**

The results reveal that the network model with a depth of 5 exhibits superior performance, albeit at the expense of the longest training time and maximum computational memory in the three models. A small server with two NVIDIA GeForce RTX 3090 GPUs with 24 G of memory was employed for this training. For the 3D U-Net model with a depth of more than 5 cannot be supported due to insufficient training memory, the 3D U-Net neural network with a depth of 5 is the commonly used and optimal choice for small servers.

## 1 Introduction

In radiotherapy, dose prediction has recently emerged as a significant area of investigation. Traditionally, radiotherapy dose prediction heavily relied on the expertise and predefined rules of plan designers, which made the process subjective and inconsistent. These limitations motivated researchers to delve into the application of deep learning techniques to enhance the accuracy and personalize the prediction of radiation dose (1).

The purpose of investigating radiotherapy dose prediction is to enhance the effectiveness of radiotherapy planning, minimize damage to healthy tissues, and improve the treatment success rate. The advancement of this technology has facilitated personalized radiotherapy treatment, allowing planners to develop more customized treatment plans based on individual patient circumstances (2). The 3D U-Net neural network structure holds tremendous significance in the field of image segmentation. This architecture, derived from the 2D U-Net, is tailored to the segmentation of volumetric images and enjoys widespread adoption (3, 4). Additionally, the 3D U-Net is specifically engineered for 3D data, rendering it an invaluable asset in the realm of dose prediction. The input of computer tomography (CT) images, organs at risk (OARs) and planning target volume (PTV) masks, and beam direction allows for the training and validation of an end-to-end network, resulting in a model that provides desired outcomes (1, 2, 5–20). However, the efficacy of the 3D U-Net model may be subject to a range of variables, including the network’s depth (2, 4, 21). In this context, “depth” refers to the quantity of both encoding and decoding layers within the network. Previous studies have examined the impact of neural network depth on image segmentation, but there is a lack of relevant research on the effect of deep learning dose prediction. Using this as a basis, we carried out a relevant study (23–26). Increased depth has the potential to capture more intricate features and achieve higher levels of accuracy. Nevertheless, more profound architectures may also lead to heightened computational complexity and lengthier training periods (21, 23–26). Consequently, thorough experimentation is often essential to determine the optimal depth for a given task.

Despite a 3D patch-based technique’s potential to alleviate large GPU memory demands from 3D volume inputs, this often sacrifices prediction accuracy. Given a 3D U-net model depth exceeding 5 is unsupported by insufficient training memory, we analyze 3, 4 and 5 depth structures. Our goal is to understand network depth’s impact on deep learning-based dose prediction for cervical cancer radiotherapy and identify optimal depth yielding the best results.

## 2 Materials and methods

### 2.1 Network architecture

The 3D U-Net is a convolutional neural network architecture that has been specifically devised for the purpose of carrying out image segmentation tasks. It is essentially an extension of the U-Net architecture and has been tailored for application in 3D image scenarios. The network is essentially made up of two indispensable components, namely, the down-sampling path (also known as the encoder path) and the up-sampling path (also referred to as the decoder path). This particular configuration is reminiscent of the shape of the letter “U”, thereby accounting for the title given to the architecture. In the network architecture of 3D U-Net, each layer of the neural network comprises two 3x3x3 convolutional operations, which are followed by Batch Normalization and the ReLU activation function. During the downsampling process, a 2x2x2 max pooling operation is utilized with a stride of 2. In the upsampling (synthesis path) process, a 2x2x2 upconvolution is applied, also with a stride of 2, and this is followed by two 3x3x3 convolutional operations and subsequent Batch Normalization.

To be more specific, the input to the 3D U-Net network is a 4-dimensional OARs mask matrix with dimensions of 128*128*128*6. The network then processes this input and generates an output dose matrix with dimensions of 128*128*128 voxel. The down-sampling pathway is composed of convolution and pooling operations that progressively diminish the dimensions of the input image whilst capturing more advanced characteristics. Conversely, the up-sampling pathway employs deconvolution operations to gradually restore the image’s size, amalgamating low-level and high-level features to yield the ultimate output.

**Figure 1:**
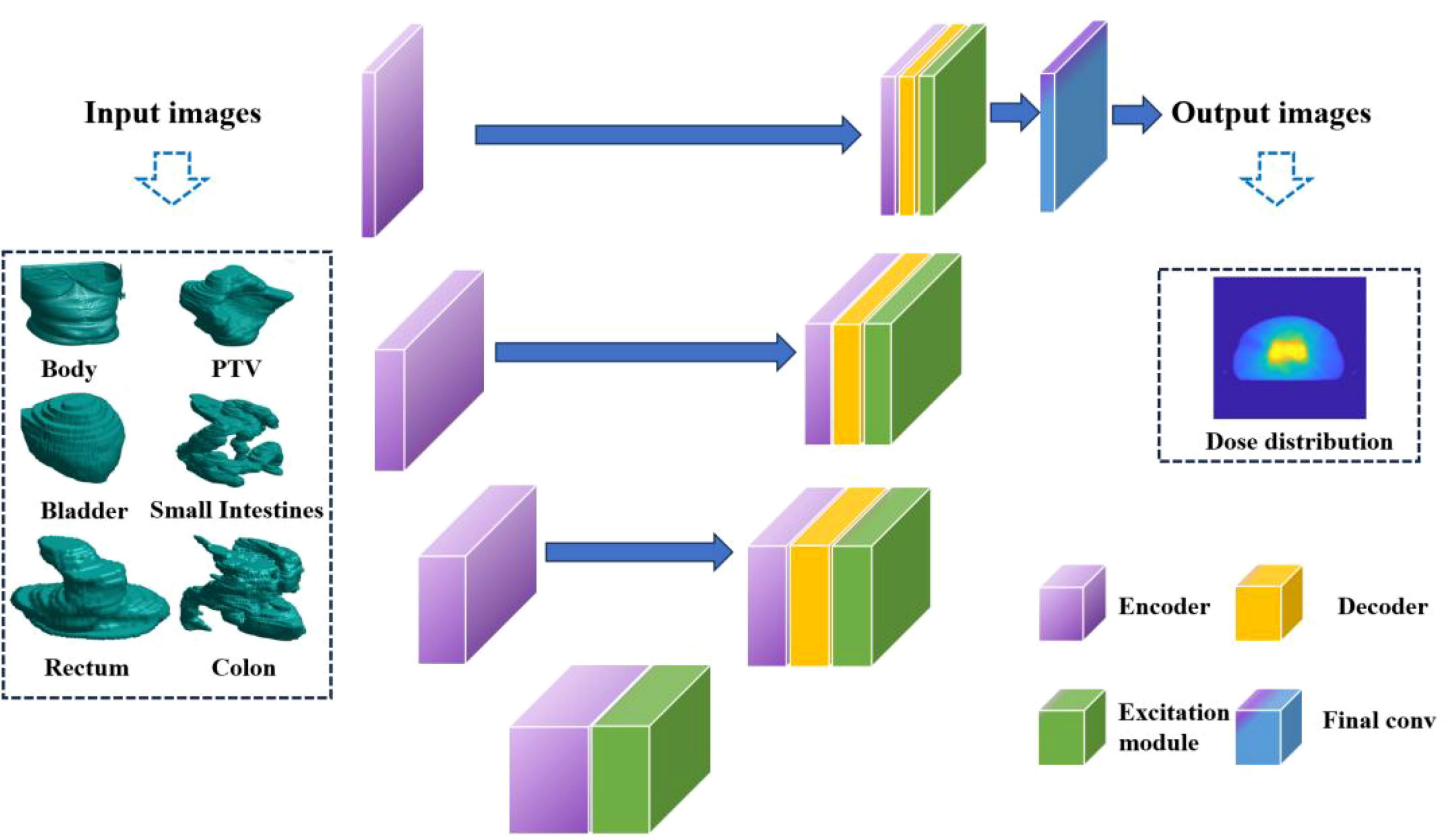
The 3D U-Net architecture.

A crucial component within the network architecture is the bottleneck layer, located in the middle. The intermediate layer plays a pivotal role in linking the down-sampling and up-sampling pathways, thereby allowing for the fusion of features and transmission of data. The utilization of this U-shaped configuration in the 3D U-Net architecture facilitates the capture of image features that span multiple scales and levels, thereby resulting in significantly enhanced accuracy in dose prediction. The 3D U-Net’s detailed structure is depicted in **Figure 1**.

The responsibility of producing pixel-level regression predictions for the input image lies with the output layer of the network. The 3D U-Net effectively extracts and integrates image features via the down-sampling and up-sampling paths and the bottleneck layer, resulting in precise dose prediction.

**Figure 2:**
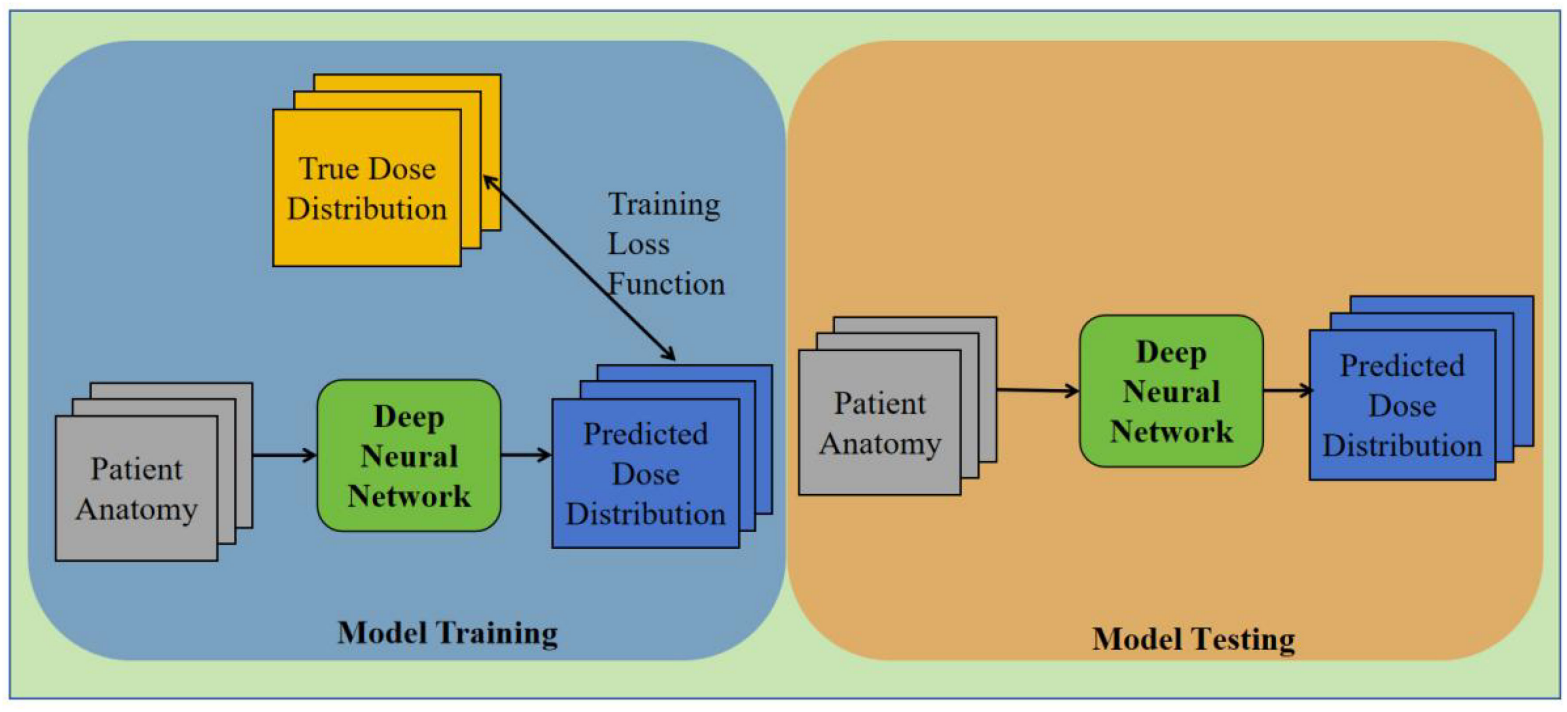
The flowchart of the training and testing process using 3D U-Net for dose prediction.

This research delves into multiple versions of the 3D U-Net architecture. A network containing 54 layers is present in the case of a depth of 3, whereas a depth of 4 has 69 layers. Moreover, the study explores a depth of 5, which elevates the total number of layers to 84. The corresponding total parameters for these structures are 19 million, 77 million, and 308.7 million, respectively. The image presented in **Figure 2** exhibits the training and testing procedures for the three network structures.

### 2.2 Data acquisition

To assess the efficacy of our approach, we employed information obtained from a cohort of 92 individuals afflicted with cervical cancer who underwent VMAT during the period spanning 2012 through 2015. The radiation dose prescribed for all participants amounted to 50.4 Gy/28 fraction. All data underwent a secondary review to remove invalid, erroneous, or incomplete data records. To better adapt the model training, dose maps were normalized. CT images may contain contrast agents and metal implants, as well as inherent noise, all of which can interfere with dose prediction accuracy. This also affects our analysis of the depth of the network versus dose prediction accuracy. All OAR masks are binary images with only two grayscale values (0, 1). These issues pose challenges during training, necessitating preliminary experiments to find optimal parameters and avoid problems. The preprocessed data were saved in the format needed for model training. The dataset was randomly split into a training set, validation set, and test set. 72 patients were randomly picked from the group for the training set, with another 10 each for the validation and test sets.

We opted to employ the 3D U-Net framework for network training, owing to its potential for dose prediction. In order to facilitate clinical applicability, the input data underwent standardization with a constant plane resolution of 1mm*1mm alongside a size of 128*128 pixel^2^. Although the axial resolution remained constant at 5mm, the number of slices exhibited variability. Consequently, we uniformly set the dimensions to 128*128*128 voxel.

Six distinct bodily organs, specifically the Body, Bladder, Rectum, PTV, Small Intestines, and Colon, were taken into account as the inputs for the network. The recorded volume information for the PTV and various OARs was meticulously documented for both the training and testing datasets, as provided in **Table 1**. It may be inferred that the training, validation, and test sets exhibit comparatively similar distributions of data. To avert over-fitting during model training, all training and validation datasets underwent geometric augmentation. In our approach to data augmentation, we utilized a variety of geometric transformation methods, which encompassed mirroring the images both horizontally and vertically, introducing random rotational changes, and applying random shifts along both the horizontal and vertical axes.

**Table 1:** PTV and OARs volume (cubic centimeter (cc)) statistics for training, validating and testing datasets (mean ± standard deviation).

	Training	Validating	Testing
Body	28028.42 ± 6524.48	35427.03 ± 7906.83	31100.48 ± 2310.39
Bladder	283.70 ± 163.54	307.08 ± 219.29	259.47 ± 85.19
Rectum	60.69 ± 27.82	78.61 ± 49.13	68.13 ± 31.75
Small Intestines	531.77 ± 217.97	591.92 ± 181.05	515.62 ± 227.19
Colon	327.68 ± 186.55	278.06 ± 97.65	416.09 ± 346.05
PTV	1082.30 ± 267.19	1124.33 ± 125.37	1148.47 ± 145.97

### 2.3 Implementation details

To obtain the best model, we selected the output model with the smallest mean variance in the validation set. The mean-variance values for the three neural network models were 29.182, 28.497, and 27.39, achieved at 13600, 9600, and 36400 iterations, respectively. The Adam optimizer was utilized in conjunction with a cost function based on mean square error (MSE) for the purpose of minimizing the loss incurred during training. The 2000 cycles that the models underwent for training had a batch size of 2 and depths of 3, 4, and 5 respectively. The training process took approximately 3102 minutes 37 seconds, 3192 minutes 41 seconds, and 3507 minutes 40 seconds for each model.

The reduction in the learning rate was determined by evaluating the number of iterations. A starting learning rate of 0.0003 was utilized with a decay coefficient of 0.95. The iterations signify the count of weight updates carried out by the model. All parameter selections have gone through extensive preliminary experiments, and the optimal parameters were determined before the training began. The implementation of our network was executed on a workstation furnished with two NVIDIA GeForce RTX 3090 GPUs.

## 3 Results

**Figure 3:**
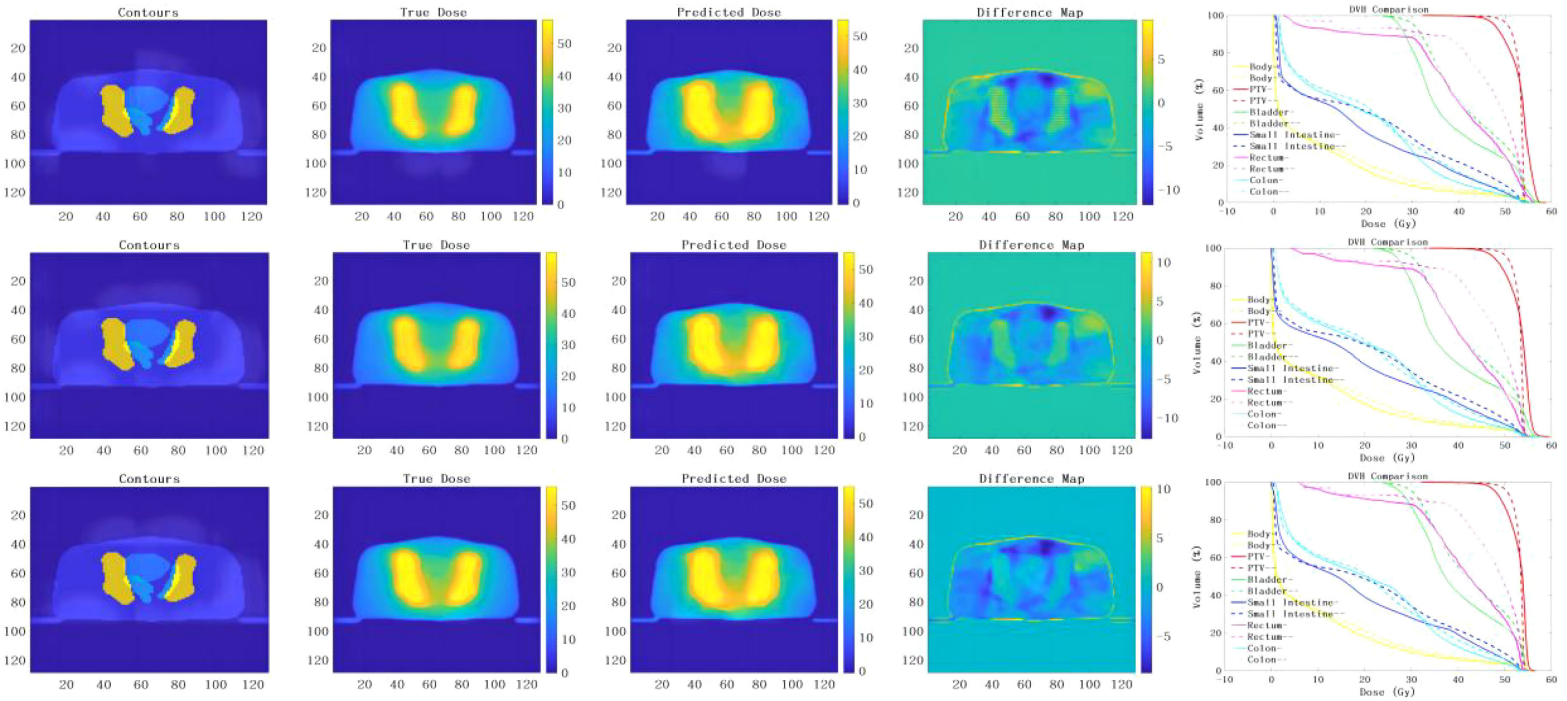
presents the prediction results obtained from the implementation of the 3D U-Net neural network models with different depths of 3, 4 and 5. The results of the models for depths 3, 4 and 5 are analyzed from top to bottom. From left to right, the input terms are contours, the true dose distribution map, the predicted dose distribution map, the difference map and the DVH comparison between the true dose and the predicted dose. (– Refers to the true dose distribution, - Refers to the predicted dose distribution).

The prediction results of the 3D U-Net neural network model with depths of 3, 4, and 5 are shown in **Figure 3**. By analyzing the difference in dose distribution, it is observed that the accuracy of PTV prediction is improved with the increase in the depth of the network. The improvements are especially evident when studying the dose volume histogram (DVH) diagram, which reveals a gradual reduction in the high dose part of the PTV area. This reduction results in a gradual overlap with the PTV, indicating more precise dose distribution prediction in PTV. Additionally, the prediction of the OARs is significantly decreased, suggesting a lower risk of adverse effects.

However, it is worth noting that the two deep network models with depths 3 and 4 were terminated prematurely due to over-fitting during training. As a result, the detailed information in the PTV was relatively lost, leading to a dose distribution prediction with relatively more discrete values. Consequently, the prediction was not as good as desired.

On the contrary, the deep neural network model with a depth of 5 had the largest network depth and underwent more extensive training without encountering over-fitting issues. This model was able to produce a dose distribution with excellent continuity, ensuring a more accurate and consistent prediction.

**Figure 4:**
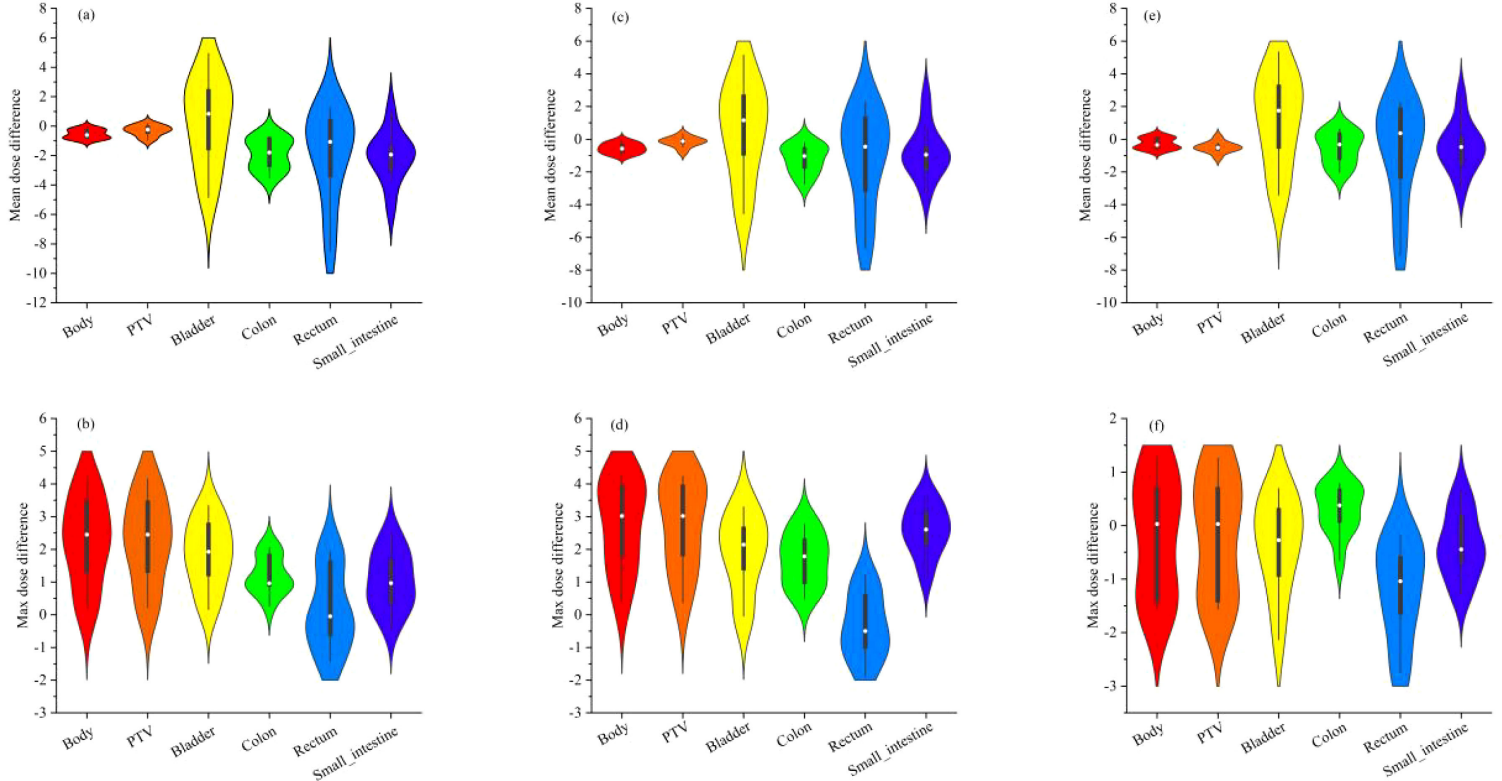
shows the absolute dose error results of 10 testing plans for mean and maximum doses in each OAR and PTV. Where **(A, B)** are the mean and maximum violin distribution of absolute dose error values for the 3 D U-Net model with depth 3, **(C, D)** are the mean and maximum violin distribution of absolute dose error values for the 3 D U-Net model with depth 4, **(E, F)** are the mean and maximum violin distribution of absolute dose error values for the 3 D U-Net model with depth 5, respectively.

**Figure 4** depicts the outcomes of the analysis of the absolute dose error, with particular emphasis on 10 treatment plans and their corresponding mean and maximum doses in each OAR and the PTV The symbols (a), (b), (c), (d), (e), and (f) demonstrate the mean and maximum dispersion of the absolute dose error values from the 3D U-Net model for the depths of 3, 4, and 5 correspondingly. Upon scrutinizing the mean absolute error of each OAR and PTV, it was observed that the Body demonstrated the most significant deviation, followed by the bladder and small intestine, and subsequently the remaining organs at risk and the PTV. Furthermore, it was discovered that the mean absolute error consistently decreased as the network depth grew.

Furthermore, analyzing the maximum absolute error in each organ and PTV revealed that the body exhibited the highest deviation, mainly noticeable in the PTV. On the other hand, the absolute dose deviation in other regions was comparatively small when compared to the analysis of mean absolute error. It was perceived that the minimum absolute error was decreased with a boost in the network depth, indicating a noteworthy improvement in the prediction accuracy of the network model.

In order to gain a comprehensive comprehension of the dose distribution within the PTV, we performed PTV dose coverage statistics for both the true and predicted dose. Specifically, we employed three evaluation indicators, namely D_98_, D_99_, and HI. D_98_ and D_99_ represent the dose obtained by 98% and 99% of the target volume, respectively, while HI is the homogeneity index. HI is calculated using the formula (D_2%_ - D_98%_) divided by D_P_. Here, D_2%_ refers to the dose received by 2% of the highest irradiated dose, and D_P_ refers to the prescribed dose. This formula for computing HI is widely used in the existing literature (22). As shown in **Table 2**, we analyzed our predictions with the ground truth in a paired two one-sided t-test. Furthermore, a Wilcoxon rank-sum test was conducted to assess the statistical differences between the three depths of network models on the feature parameters D_98_, D_99_, and HI. The results demonstrated no statistically significant differences (p > 0.05) between the depth 3 and depth 4 models for both D_98_ and D_99_, as well as HI. However, a statistically significant difference (p < 0.05) was observed between the depth 3 and depth 5 models, and between the depth 4 and depth 5 models. In terms of numerical performance, the depth 5 model exhibited the most superior results.

**Table 2:** PTV dose coverage statistics for true and predicted doses (mean ± standard deviation).

	True dose	Predicted dose	True-prediction	P value
D_98__depth3	47.00 ± 0.91	45.67 ± 0.36	1.33 ± 0.55	2.57e-3
D_98__depth4	47.00 ± 0.91	45.64 ± 0.33	1.36 ± 0.58	2.86e-2
**D_98__depth5**	**47.00 ± 0.91**	**46.00 ± 0.68**	**1.00 ± 0.23**	**3.64e-2**
D_98__Pvalue Depth(3vs4/3vs5/4vs5)		0.85/0.02/0.01		
D_99__depth3	45.28 ± 1.20	44.12 ± 0.44	1.16 ± 0.76	**2.51e-2**
D_99__depth4	45.28 ± 1.20	44.18 ± 0.39	1.1 ± 0.81	2.85e-2
**D_99__depth5**	**45.28 ± 1.20**	**44.96 ± 0.48**	**0.32 ± 0.72**	**0.43**
D_99__P value Depth(3vs4/3vs5/4vs5)		0.76/1.5e-3/2.9e-3		
Homogeneity_depth3	0.17 ± 0.03	0.22 ± 0.01	-0.05 ± 0.02	1.26e-3
Homogeneity_depth4	0.17 ± 0.03	0.21 ± 0.01	-0.04 ± 0.02	6.36e-3
**Homogeneity_depth5**	**0.17 ± 0.03**	**0.19 ± 0.01**	**-0.02 ± 0.02**	**0.16**
Homogeneity_P value Depth(3vs4/3vs5/4vs5)		0.059/5.8e-4/2.8e-3		

The bolded values indicate the best performance.

**Table 3** presents the mean and maximum dose’s mean and standard deviation across the OARs and the PTV for the ten test plans. The outcomes suggest that the projected dose is accurate in comparison to the authentic dose. As illustrated in **Table 3**, we conducted a paired two-sample one-sided t-test to evaluate the accuracy of our predictions in comparison to the ground truth. Additionally, a Wilcoxon rank sum test was conducted to assess statistical differences between the three deep network models on different OARs and PTV. No statistically significant differences were observed between the OARs with regard to mean dose (p > 0.05). However, a statistically significant difference was identified between the PTV for depths 4 and 5 (p < 0.05). With regard to the maximum dose, a statistically significant difference (p < 0.05) was observed between the OARs and PTV in all three models, with the exception of the bladder and colon. In terms of numerical performance, the depth 5 model exhibited the most superior results.

**Table 3:** The results of 10 testing plans for mean and maximum doses in each OAR and PTV (mean ± standard deviation).

	Mean dose					Maximum dose				
	True	Depth3/ P value	Depth4/ P value	Depth5/ P value	P ValueDepth(3vs4/3vs5/4vs5)	True	Depth3/ P value	Depth4/ P value	Depth5/ P value	P Value Depth (3vs4/3vs5/4vs5)
Body	10.06 ± 0.99	9.51 ± 0.83/ 4.1e-4	9.50 ± 0.80/ 3.5e-4	9.79 ± 0.81/ 4.1e-2	0.91/ 0.48/ 0.44	56.81 ± 1.17	59.08 ± 0.56/ 4.8e-4	59.57 ± 0.45/ 1.5e-4	56.63 ± 0.32/ 0.62	0.04/ 1.08e-5/1.08e-5
Bladder	41.81 ± 1.77	42.12 ± 2.59/ 0.76	42.40 ± 2.65/ 0.55	43.05 ± 2.38/ 0.20	0.53/ 0.12/ 0.22	56.00 ± 1.02	57.84 ± 0.17/ 3.4e-4	57.89 ± 0.60/ 7.0e-4	55.65 ± 0.38/ 0.25	0.68/ 1.08e-5/1.08e-5
Rectum	42.70 ± 3.91	40.74 ± 3.90/ 0.08	41.53 ± 3.67/ 0.24	41.96 ± 3.84/ 0.48	0.58/ 0.48/ 0.74	56.07 ± 0.98	56.21 ± 0.45/ 0.72	55.71 ± 0.32/ 0.29	54.85 ± 0.66/ 2.4e-3	0.01/ 2.1e-4/5.2e-3
Small Intestines	23.77 ± 5.59	21.68 ± 6.17/ 4.2e-3	22.95 ± 6.33/ 0.14	23.32 ± 6.13/ 0.38	0.68/ 0.58/ 0.74	55.55 ± 0.85	56.59 ± 0.56/ 5.4e-3	58.12 ± 0.68/ 2.4e-6	55.17 ± 0.50/ 0.09	2.1e-4/ 1.3e-4/ 1.08e-5
Colon	25.02 ± 4.72	23.12 ± 4.48/ 3.5e-4	23.81 ± 4.85/ 1.9e-3	24.48 ± 4.75/ 0.12	0.74/ 0.48/ 0.63	55.06 ± 0.60	56.27 ± 0.30/ 1.1e-4	56.74 ± 0.69/ 1.1e-4	55.36 ± 0.74/ 0.07	0.08/ 2.9e-3/ 7.3e-4
PTV	53.38 ± 0.33	53.08 ± 020/ 0.027	53.22 ± 0.25/ 0.13	52.89 ± 0.31/ 1.2e-3	0.35/ 0.05/ 0.01	56.81 ± 1.17	59.08 ± 0.56/ 4.8e-4	59.57 ± 0.45/ 1.5e-4	56.63 ± 0.32/ 0.62	0.04/ 1.1e-5/ 1.1e-5

Regarding the PTV, the D_98_ value from the model with a depth of three is 47.00 ± 0.91, while the projected dose has a mean value of 45.67 ± 0.36. The evidence implies that the anticipated dosage is slightly below the authentic amount, with a mean variance of 1.33 ± 0.55. Likewise, for models with depths 4 and 5, the anticipated doses are also marginally lower than the authentic doses, with mean differences of 1.36 ± 0.58 and 1.00 ± 0.23, respectively. The D_99_ follows a similar pattern, with the projected dose being marginally lower than the authentic dose.

The HI, which quantifies the consistency of the dose distribution within the PTV, exhibits negative values across all three models, thus signifying that the anticipated doses demonstrate greater variability when compared to their actual counterparts. It is noteworthy that the increase in network depth results in a decrease in HI values, suggesting that the dose distribution tends towards uniformity as the neural network’s depth increases.

These results suggest that the predicted doses from the 3D U-Net neural network models are slightly lower than the true doses within the PTV. However, the dose deviations are relatively small and decrease with the increase in the network depth, indicating improved prediction accuracy.

## 4 Discussion

The 3D U-Net deep neural network structure is widely utilized for dose prediction in radiotherapy. However, the attention to the network depth and its impact on the accuracy and robustness of dose prediction remains inadequate. In this study, we explored the effects of network depth on dose prediction by training and testing three different 3D U-Net structures with depths of 3, 4, and 5 using data from 92 cervical cancer patients who underwent Volumetric Modulated Arc Therapy (VMAT). Our statistical analysis revealed significant differences in maximum dose for various OARs and PTV parameters (D_98_, D_99_, and HI) across depth 4 and depth 5 models. Deeper models generally showed smaller maximum dose and better uniformity. These findings inform subsequent model depth selection, suggesting a deeper network structure within constraints of limited training data, hardware capabilities, and reasonable hyperparameter and data augmentation settings.

Although deeper networks may have more parameters and be prone to over-fitting, they can capture more relevant features of the radiation dose and aid in accurate prediction. Additionally, the network depth affects training time, computational resources, and potential issues like gradient disappearance or explosion. Overall, the selection of network depth involves balancing various factors and considering the trade-offs between accuracy, robustness, and resource constraints.

While other deep learning algorithms like GAN and diffusion models with similar U-net structures can also be used for dose prediction, the impact of network depth is a common issue. Taking the 3D U-net structure as an example supports our conjecture (1, 15–17). The impact of network depth on radiotherapy dose prediction requires careful evaluation and optimization for specific tasks and datasets. Adding depth to a network significantly increases complexity and enhances information understanding and processing, improving prediction accuracy. However, deeper networks require more GPU memory and training time. Choosing the appropriate depth is crucial, guided by task-specific testing and evaluation. Data enhancement via geometric techniques is beneficial but challenging due to memory and computational power requirements. Inadequate data augmentation often leads to overfitting, emphasizing the importance of selecting appropriate network depth. Limitations like the inability to test 3D U-nets beyond depth 5 due to GPU memory constraints further complicate matters. It’s worth noting a 128x128x128 image is saturated for 3D U-net testing at depth 5.

## 5 Conclusion

The study focused on assessing the influence of neural network models with varying depths in predicting radiation dose for cervical cancer. The results reveal that the network model with a depth of 5 exhibits superior performance, albeit at the expense of the longest training time and maximum computational memory in the three models. A small server with two NVIDIA GeForce RTX 3090 GPUs with 24 G of memory was employed for this training. The 3D U-net neural network model with a depth of more than 5 cannot be supported due to insufficient training memory, while the comparative analysis of the models with depths of 3, 4, and 5 yields the best performance for the model with a depth of 5. Therefore, for the training of small servers, the 3D U-Net neural network model with depth 5 is the commonly used and optimal choice. This informs subsequent model depth selection, suggesting a deeper network structure within constraints of limited training data, hardware capabilities, and reasonable hyperparameter and data augmentation settings.

## Data Availability

The original contributions presented in the study are included in the article/supplementary material. Further inquiries can be directed to the corresponding author.

## References

[1] Wang M Zhang Q Lam S Cai J Yang R. A review on application of deep learning algorithms in external beam radiotherapy automated treatment planning. Front Oncol. (2020) 10:580919. doi: 10.3389/fonc.2020.580919 33194711 PMC7645101

[2] Qilin Z Peng B Ang Q Weijuan J Ping J Hongqing Z. The feasibility study on the generalization of deep learning dose prediction model for volumetric modulated arc therapy of cervical cancer. J Appl Clin Med Phys vol. (2022) 23:6. doi: 10.1002/acm2.13583 PMC9195039 35262273

[3] Zhang S Wang H Tian S Zhang X Li J Lei R. A slice classification model-facilitated 3D encoder-decoder network for segmenting organs at risk in head and neck cancer. J Radiat Res vol. (2021) 62:94–103. doi: 10.1093/jrr/rraa094 PMC7779351 33029634

[4] iek zgün Abdulkadir A Lienkamp SS Brox T Ronneberger O. 3D U-net: learning dense volumetric segmentation from sparse annotation. Cham: Springer (2016).

[5] Ma J Nguyen D Bai T Folkerts M Jia X Lu W. A feasibility study on deep learning-based individualized 3D dose distribution prediction. ” Med Phys vol. (2021) 48:4438–47. doi: 10.1002/mp.15025 PMC8842508 34091925

[6] Barragán-Montero AM Nguyen D Lu W Lin MH Norouzi-Kandalan R Geets X. Three-dimensional dose prediction for lung IMRT patients with deep neural networks: robust learning from heterogeneous beam configurations. Med Phys vol. (2019) 46:3679–91. doi: 10.1002/mp.13597 31102554

[7] Zhang B Babier A Chan T Ruschin M. 3D dose prediction for Gamma Knife radiosurgery using deep learning and data modification. Physica medica: PM: an Int J devoted to Appl Phys to Med biology: Off J Ital Assoc Biomed Phys (AIFB). (2023) 106:102533. doi: 10.1016/j.ejmp.2023.102533 36724551

[8] Fan J Wang J Chen Z Hu C Zhang Z Hu W. Automatic treatment planning based on three-dimensional dose distribution predicted from deep learning technique. Med Phys. (2019) 46:370–81. doi: 10.1002/MP.13271 30383300

[9] Svecic A Roberge D Kadoury S. Prediction of inter-fractional radiotherapy dose plans with domain translation in spatiotemporal embeddings. Med Image Anal. (2020) 64:101728. doi: 10.1016/j.media.2020.101728 32622121

[10] Wang J Hu J Song Y Wang Q Zhang X Bai S. VMAT dose prediction in radiotherapy by using progressive refinement UNet. Neurocomputing. (2022) 488:528–39. doi: 10.1016/j.neucom.2021.11.061

[11] Ma M Buyyounouski K Vasudevan V Xing L Yang Y. Dose distribution prediction in isodose feature-preserving voxelization domain using deep convolutional neural network. Med Phys. (2019) 46:2978–87. doi: 10.1002/MP.13618 31112305

[12] Kearney V Chan JW Haaf S Descovich M Solberg TD. DoseNet: A volumetric dose prediction algorithm using 3D fully-convolutional neural networks. Phys Med Biol. (2018) 63:235022. doi: 10.1088/1361-6560/AAEF74 30511663

[13] Castriconi R Fiorino C Passoni P Broggi S di Muzio NG Cattaneo GM. Knowledge-based automatic optimization of adaptive early-regression-guided VMAT for rectal cancer. Physica Med. (2020) 70:58–64. doi: 10.1016/j.ejmp.2020.01.016 31982788

[14] Liu H Xing L. Isodose feature-preserving voxelization (IFPV) for radiation therapy treatment planning. Med Phys. (2018) 45:3321–9. doi: 10.1002/MP.12977 PMC6041150 29772065

[15] Wen L Xiao J Zeng J Zu C Wu X Zhou J. Multi-level progressive transfer learning for cervical cancer dose prediction. Pattern Recognit. (2023) 141. doi: 10.1016/j.patcog.2023.109606

[16] Jiao Z Peng X Wang Y Xiao J Nie D Wu X. TransDose: Transformer-based radiotherapy dose prediction from CT images guided by super-pixel-level GCN classification. Med Image Anal. (2023) 89:102902. doi: 10.1016/j.media.2023.102902 37482033

[17] Song Y Hu J Liu Y Hu H Huang Y Bai S. Dose prediction using a deep neural network for accelerated planning of rectal cancer radiotherapy. Radiother Oncol. (2020) 149:111–6. doi: 10.1016/J.RADONC.2020.05.005 32416279

[18] Jia Q Zheng C Li Y Guo F Zhou L Song T. A predicted three-dimensional dose sequence based treatment planning optimization method for gynecologic IMRT. Med Eng Phys. (2023) 118:104011. doi: 10.1016/j.medengphy.2023.104011 37536834

[19] Oh K Gronberg MP Netherton TJ Sengupta B Cardenas CE Court LE. A deep-learning-based dose verification tool utilizing fluence maps for a cobalt-60 compensator-based intensity-modulated radiation therapy system. Phys Imaging Radiat Oncol. (2023) 26:100440. doi: 10.1016/j.phro.2023.100440 37342210 PMC10277917

[20] Li F Niu S Han Y Zhang Y Dong Z Zhu J. Multi-stage framework with difficulty-aware learning for progressive dose prediction. Biomed Signal Process Control. (2023) 82:104541. doi: 10.1016/j.bspc.2022.104541

[21] Benkendorf DJ Hawkins CP. Effects of sample size and network depth on a deep learning approach to species distribution modeling. Ecol Inf. (2020) 60:101137. doi: 10.1016/J.ECOINF.2020.101137

[22] Yan L Xu Y Chen X Xie X Liang B Dai J. A new homogeneity index definition for evaluation of radiotherapy plans. J Appl Clin Med Phys vol. (2019) 20:11. doi: 10.1002/acm2.12739 PMC6839365 31605454

[23] Jena B Jain S Nayak GK Saxena S. Analysis of depth variation of u-net architecture for brain tumor segmentation. Multimed Tools Appl. (2023) 82:10723–43. doi: 10.1007/s11042-022-13730-1

[24] Celaya A Actor JA Muthusivarajan R Gates E Chung C Schellingerhout D. Pocketnet: a smaller neural network for medical image analysis. IEEE Trans Med Imaging. (2023) 42:1172–84. doi: 10.1109/TMI.2022.3224873 PMC10882585 36427285

[25] Gay SS Kisling KD Anderson BM Zhang L Rhee DJ Nguyen C. Identifying the optimal deep learning architecture and parameters for automatic beam aperture definition in 3d radiotherapy. J Appl Clin Med Phys. (2023) 24:e14131. doi: 10.1002/acm2.14131 37670488 PMC10691634

[26] Jena B Digdarshi D Paul S Nayak GK Saxena S. Effect of learning parameters on the performance of the u-net architecture for cell nuclei segmentation from microscopic cell images. Microscopy (Oxf). (2023) 72:249–64. doi: 10.1093/jmicro/dfac063 36409001

